# Single cell neuro-sensory dynamics: Ca^2+ ^chemoreceptor-guided sea urchin sperm motility

**DOI:** 10.1186/1471-2202-14-S1-P173

**Published:** 2013-07-08

**Authors:** J Nathan Kutz, Lisa J Burton, Yasmeen Hussain, Jeff Riffell, Jeffrey S Guasto, Roman Stocker, AE Hosoi

**Affiliations:** 1 Department of Applied Mathematics, University of Washington, Seattle, WA, 98195, USA; 2 Department of Mechanical Engineering, Massachusetts Institute of Technology, Cambridge, MA, 02139, USA; 3 Department of Biology, University of Washington, Seattle, WA, 98195, USA; 4 Civil and Environmental Engineering, Massachusetts Institute of Technology, Cambridge, MA, 02139, USA

## 

Neuro-sensory systems are critical for integrating environmental stimuli and providing a framework for resolving decision-making tasks. Remarkably, the molecular mechanisms mediating transduction of sensory information in neurons are also found in other cellular tissues, including sperm. One mechanism facilitating such behavior is a sperm's ability to perform chemotaxis to egg-derived compounds, a phenomenon in which sperm orient to an attractant gradient around an egg. The ensuing motility of the sperm cell is driven by attractants binding to olfactory G-coupled receptor proteins - located on the cell membrane surface - that elicits a signal transduction cascade similar to the one that occurs in the mammalian olfactory neurons located in the nasal epithelium. Receptor binding of the egg-derived compound elicits a cyclic-AMP transduction cascade that produces localized, cytosolic Ca^2+ ^responses in the cell body. This response in turn controls flagellar beating via Ca^2+ ^-sensitive axonemal motor proteins [1]. Thus through a process of chemo-sensory integration, analogous to that which occurs in olfactory receptor neurons, the sperm cell is driven to interact with its fluid environment for the specific goal of fertilization.

Critical to the success of the fertilization process is the sperm's ability to swim to its targeted destination. The swimming dynamics itself represent a complicated interaction between the flagella and the fluid environment in which it is immersed. Quantifying the low Reynolds number swimming dynamics requires that the flagellar shape be accurately modeled as a function of both space and time. Despite the complex kinematics, dimensionality reduction techniques reveal low-dimensional dynamics that accurately represent the swimming dynamics with only a few parameters. Specifically, a few optimal modes can model and predict the swimming speed and trajectory of the cell [2]. In our work, we show that such a model reduction of the swimming sperm cell can be directly integrated with a model of the Ca^2+ ^dynamics in the cell body which controls the beating (driving) of the flagella, thus providing a comprehensive model of the neuro-sensory input-output response of the sperm cell. The theoretical model developed is compared to experimental findings using gametes from the sea urchins *Arbacia punctulata *and *Lytechinus pictus*. Using a microfluidic laminar-flow device, a chemical gradient is established with known chemoattractants. Sperm placed within the device are simultaneously imaged for motility, orientation, and calcium responses under simulated hydrodynamic conditions. The experimental findings support the modeling efforts and highlight the underlying neuro-sensory processing responsible for driving sperm motility.

**Figure 1:**
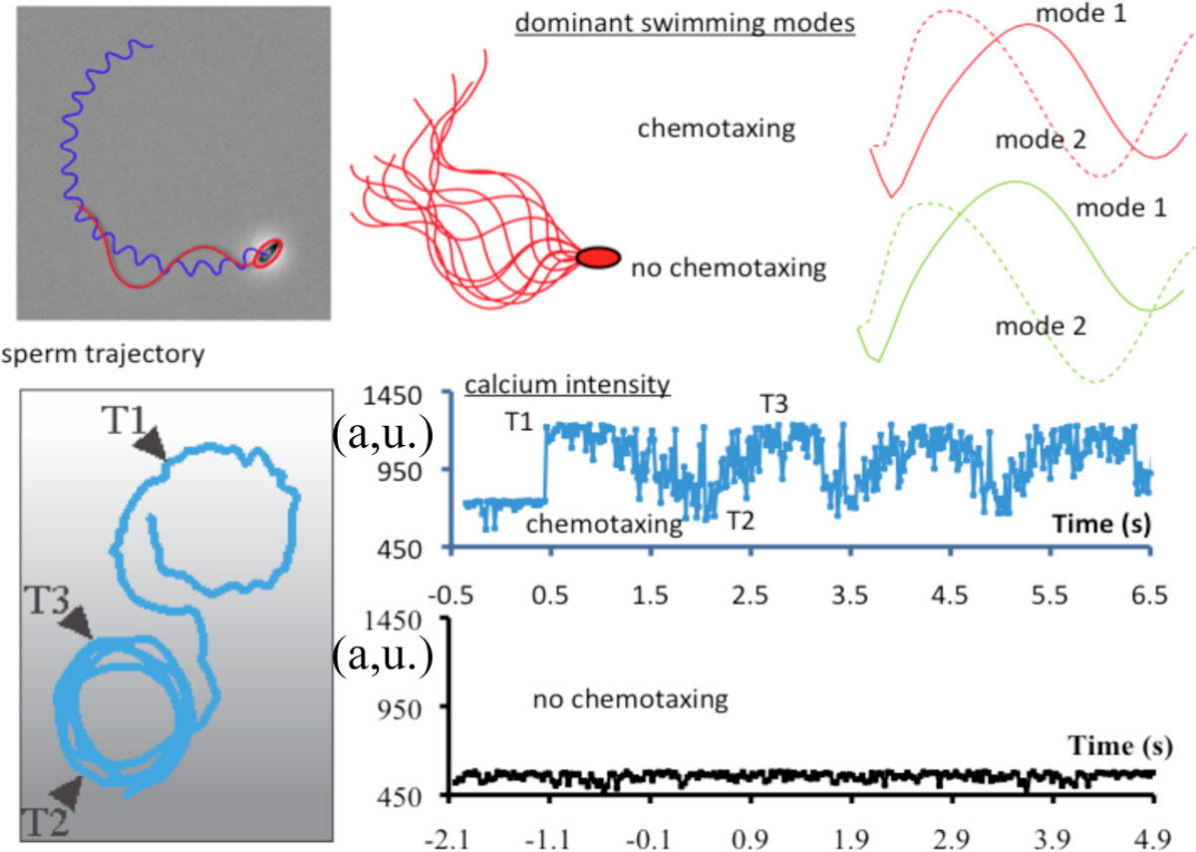
**Trajectory of sperm under chemotaxis along with an illustration of the modal shapes and calcium dynamics associated with swimming for both chemo- and non-chemotaxing behavior**.
